# Ross Sea Mollusca from the Latitudinal Gradient Program: R/V *Italica* 2004 Rauschert dredge samples

**DOI:** 10.3897/zookeys.341.6031

**Published:** 2013-10-07

**Authors:** Claudio Ghiglione, Maria Chiara Alvaro, Huw J. Griffiths, Katrin Linse, Stefano Schiaparelli

**Affiliations:** 1 Department of Earth, Environmental and Life Sciences (DISTAV), University of Genoa, Genoa, Italy; 2 Italian Antarctic National Museum (MNA), University of Genoa, Genoa, Italy; 3 British Antarctic Survey (BAS), Cambridge, United Kingdom

**Keywords:** Antarctica, Ross Sea, Mollusca, Gastropoda, Bivalvia, Monoplacophora, Aplacophora, Polyplacophora, Scaphopoda, Italica 2004, Rauschert dredge, Latitudinal Gradient Program

## Abstract

Information regarding the molluscs in this dataset is based on the Rauschert dredge samples collected during the Latitudinal Gradient Program (LGP) on board the R/V “*Italica*” in the Ross Sea (Antarctica) in the austral summer 2004. A total of 18 epibenthic dredge deployments/samplings have been performed at four different locations at depths ranging from 84 to 515m by using a Rauschert dredge with a mesh size of 500μm. In total 8,359 specimens have been collected belonging to a total of 161 species. Considering this dataset in terms of occurrences, it corresponds to 505 discrete distributional records (incidence data). Of these, in order of abundance, 5,965 specimens were Gastropoda (accounting for 113 species), 1,323 were Bivalvia (accounting for 36 species), 949 were Aplacophora (accounting for 7 species), 74 specimens were Scaphopoda (3 species), 38 were Monoplacophora (1 species) and, finally, 10 specimens were Polyplacophora (1 species). This data set represents the first large-scale survey of benthic micro-molluscs for the area and provides important information about the distribution of several species, which have been seldom or never recorded before in the Ross Sea. All vouchers are permanently stored at the Italian National Antarctic Museum (MNA), Section of Genoa, enabling future comparison and crosschecking. This material is also currently under study, from a molecular point of view, by the barcoding project “BAMBi” (PNRA 2010/A1.10).

## Purpose

This dataset is about the mollusc samples obtained in the framework of the 2004 voyage of the RV “*Italica*”, under the Latitudinal Gradient Program (LGP), by deploying a fine-mesh dredge (Rauschert dredge). This collection is now part of the Italian National Antarctic Museum (MNA, Section of Genova) and is published with the aim of increasing the knowledge of the distribution of mollusc species in the Ross Sea. The dataset is also the first Italian contribution to ANTABIF based on materials stored at the MNA.

## Project details

**Project title:** Latitudinal Gradient Program (LGP) R/V “Italica” voyage 2004 - Mollusca.

**Curator and Promoter:** Stefano Schiaparelli.

**Personnel:** Claudio Ghiglione, Maria Chiara Alvaro, Huw J. Griffiths, Katrin Linse.

**Funding:** This study is part of the Project 2002/8.6 (“The coastal ecosystem of Victoria Land Coast: distribution and structure along a latitudinal gradient”) and of the Project 2010/A1.10 (Barcoding of Antarctic Marine Biodiversity, BAMBi) funded by the Italian National Antarctic Research Program (PNRA). Vouchers are maintained at the Italian National Antarctic Museum (MNA), Section of Genoa.

**Study area description:** This dataset lists the species that have been collected by deploying for the first time a Rauschert dredge in the Ross Sea (Rehm et al. 2006). Samples were obtained during the Austral summer 2004 in the framework of the 19^th^ PNRA Antarctic expedition, on board the R/V *“Italica”*. The study area was the continental shelf along the latitudinal transect comprised between Cape Adare (~71°S) and Terra Nova Bay (~75°S) (Fig. 2). The Rauschert dredge was deployed in its ‘standard’ structure, i.e. having a mesh size of 500µm and with an opening of 0.5 m (Lörz et al. 1999). The dredge has been towed at a mean velocity of 1 knot to collect benthic samples at eighteen stations, comprised between 84 and 515m of depth. More details about sampling stations are reported in Rehm et al. (2006).

**Design description:** In the past decade, the Ross Sea has been the area studied by the Latitudinal Gradient Program (LGP; www.lgp.aq) which aimed at: i) understanding the complex ecosystems that exist along the Victoria Land coast; and ii) determining the effects of environmental change on these ecosystems; iii) maximising the transfer of information and ideas, by utilising joint logistic facilities. To achieve these targets, two temporally parallel research voyages took place during the Austral summer 2004: one on board the Italian R/V “*Italica*” and one on board the R/V “*Tangaroa*” (“BioRoss” voyage, TAN0402) organized, respectively, by NIWA (National Institute of Water and Atmospheric Research, Wellington) and PNRA (Antarctic National Research Project).

**Figure 1.**
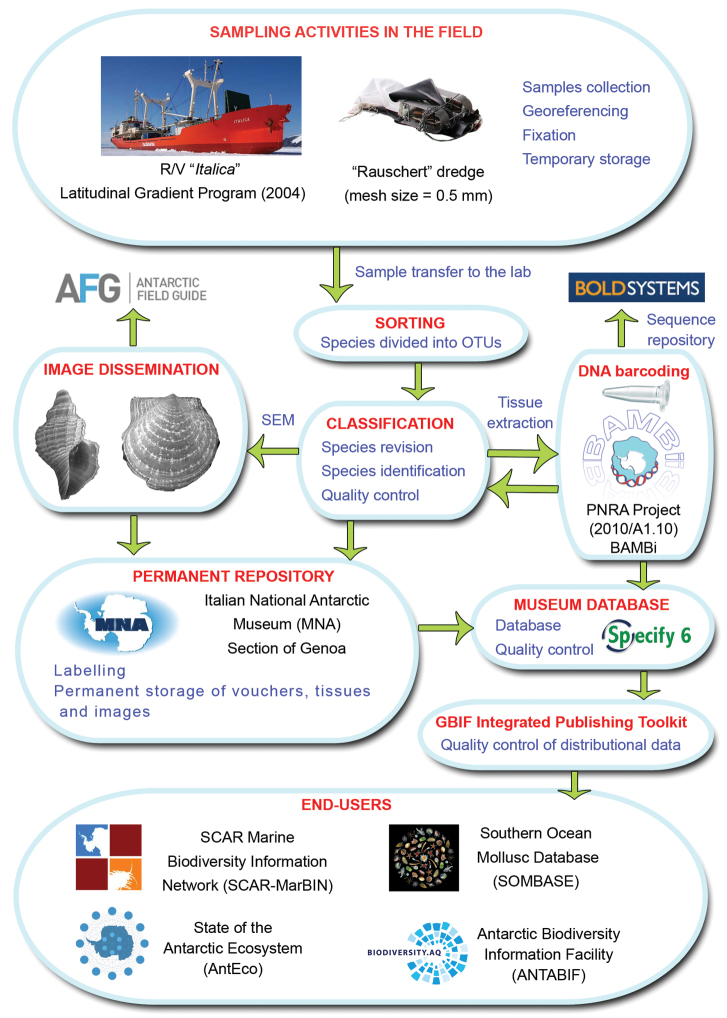
Flowchart depicting major steps in dataset development and publishing.

In the field, samples were collected by using a Rauschert dredge with a mesh size of 500μm (Lörz et al. 1999). Samples were fixed on board in precooled Ethanol in order to have material suitable for genetic studies. Sorting and classification was performed at the Italian National Antarctic Museum (MNA), Section of Genoa, where all the samples were acquired as permanent vouchers. The digital and SEM images of the mollusc species studied will be made available through the ANTABIF Antarctic Field Guide Project (http://afg.biodiversity.aq/about). The LGP contributed to the SCAR biology programme Evolution and Biodiversity in the Antarctic (EBA) and now to the SCAR programme State of the Antarctic Ecosystem (AntEco). The dataflow is illustrated in Figure 1.

**Figure 2.**
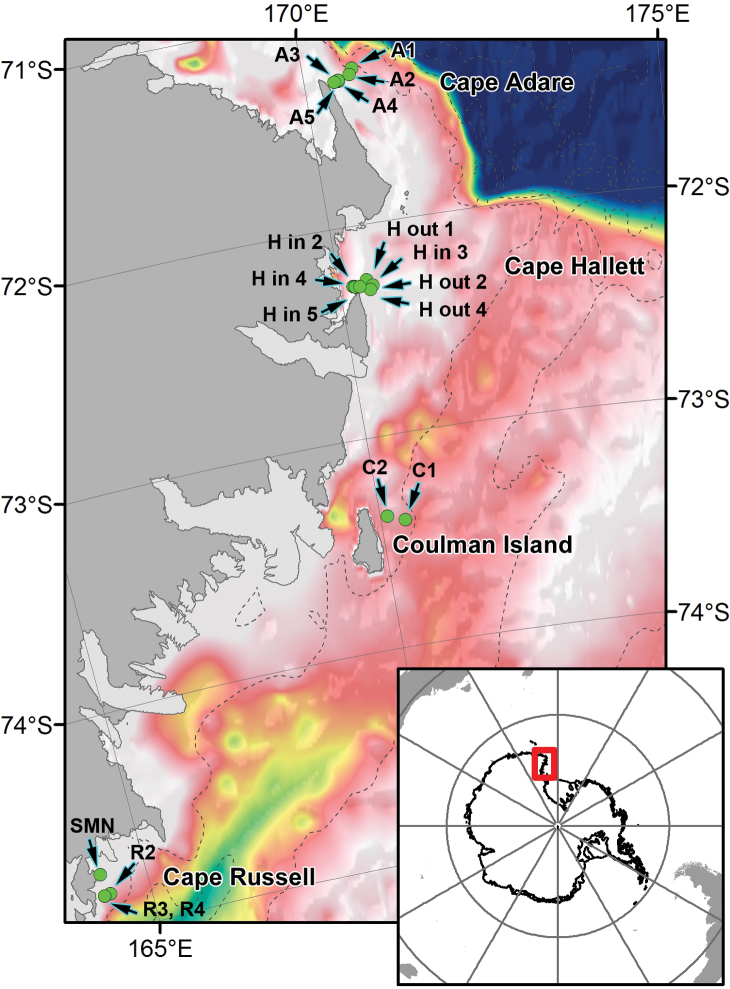
Map of sampling stations.

## Methods

### Method step description

The material was collected during the R/V “*Italica*” 2004 LGP Expedition by Dr. Peter Rehm (see Rehm et al. 2006) under the framework of the PNRA Project 2002/8.6. In the specific, the Rauschert samples were preserved immediately in pre-cooled 90% ethanol and kept in -25°C for later DNA extraction. Sorting of molluscs was performed at the Italian National Antarctic Museum. Taxonomic identification was performed at the Italian Antarctic National Museum (MNA, Section of Genoa) and at the British Antarctic Survey (BAS) laboratories. All living specimens were sorted under a stereomicroscope and, whenever possible, classified down to the specific level. More minute species were photographed at ESEM (Leo Stereoscan 440) facility at DISTAV. Dead shells have not been taken into account in the present study.

The present molluscs dataset has been formatted in order to fulfil the standards (Darwin Core) required by the OBIS scheme (http://iobis.org/data/schema-and-metadata) according the SCAR-MarBIN Data Toolkit (available at http://www.scarmarbin.be/documents/SM-FATv1.zip). The dataset was uploaded in the ANTOBIS database (the geospatial component of SCAR-MarBIN) and added to SOMBASE (Southern Ocean Mollusc Database, www.antarctica.ac.uk/sombase). SOMBASE generated initial core data system upon which SCAR’s Marine Biodiversity Information Network (SCAR-MarBIN) was built. Taxonomy was matched against the Register of Antarctic Marine Species, using the Taxon Match tool (http://www.scarmarbin.be/rams.php?p=match). Data from both the R/V “*Italica*” and the R/V “*Tangaroa*” (“BioRoss”, TAN0402) voyages were published in Schiaparelli et al. (2006). A detailed analysis of the distribution of mollusc species sampled by the Rauschert dredge as well as the illustration of all new records for the Ross Sea is in Ghiglione et al. (submitted). The dataflow is illustrated in Figure 1.

**Study extent**: This dataset lists the species that have been collected by deploying for the first time a Rauschert dredge in the Ross Sea (Rehm et al. 2006). Samples were obtained during the Austral summer 2004 in the framework of the 19th PNRA Antarctic expedition, on board the R/V “*Italica*”. The study area was the continental shelf along the latitudinal transect comprised between Cape Adare (~71°S) and Terra Nova Bay (~75°S). On the whole,eighteen stations, comprised between 84 and 515m of depth, were sampled.

**Sampling description:** Sampling activities were done in four main areas of the Ross Sea: Cape Adare, Cape Hallett, Coulman Island, Cape Russell (Fig. 2). The eighteen Rauschert dredge samples were obtained at Cape Adare (five sampling sites: A1, A2, A3, A4, A5), at Cape Hallett (seven sampling sites: H out 1, H out 2, H out 4, H in 2, H in 3; H in 4, H in 5), at Coulmann Island (two sites: C1, C2) and at Cape Russell (four sites: SMN, R2, R3, R4) (Rehm et al. 2006).

**Quality control description:** Specimens were classified at the lowest possible taxonomic level. A 18% of species were classified at the or above the generic level due to an uncertainty about their status. The same was for another 6% of species that potentially represent new species. Although these taxa will require further studies (e.g. morphological and genetic, currently underway) these have been included in this dataset as they could be clearly distinguished during sorting activities and were therefore considered as morphospecies. During all the phases of sorting, classification and storage of samples at the Italian National Antarctic Museum, quality controls and data cleaning have been undertaken at various steps in order to produce quality data and make consistent cross-references between the database and samples’ labels. The MNA uses an SQL-based database (Specify 6) to manage its collections and link all the data (photos, sequences, etc.) to the physical samples. Georeferencing on board the R/V “*Italica*” is based on the interpolation of GPS satellite receivers (models 3S Navigation and Glonass ASHTECH GG24) and a gyrocompass. Station coordinates and sampling events were recorded during sampling activities through the “*Italica*” NetNav WEB system, which is based on the above GPS systems.

## Taxonomic coverage

**Figure 3.**
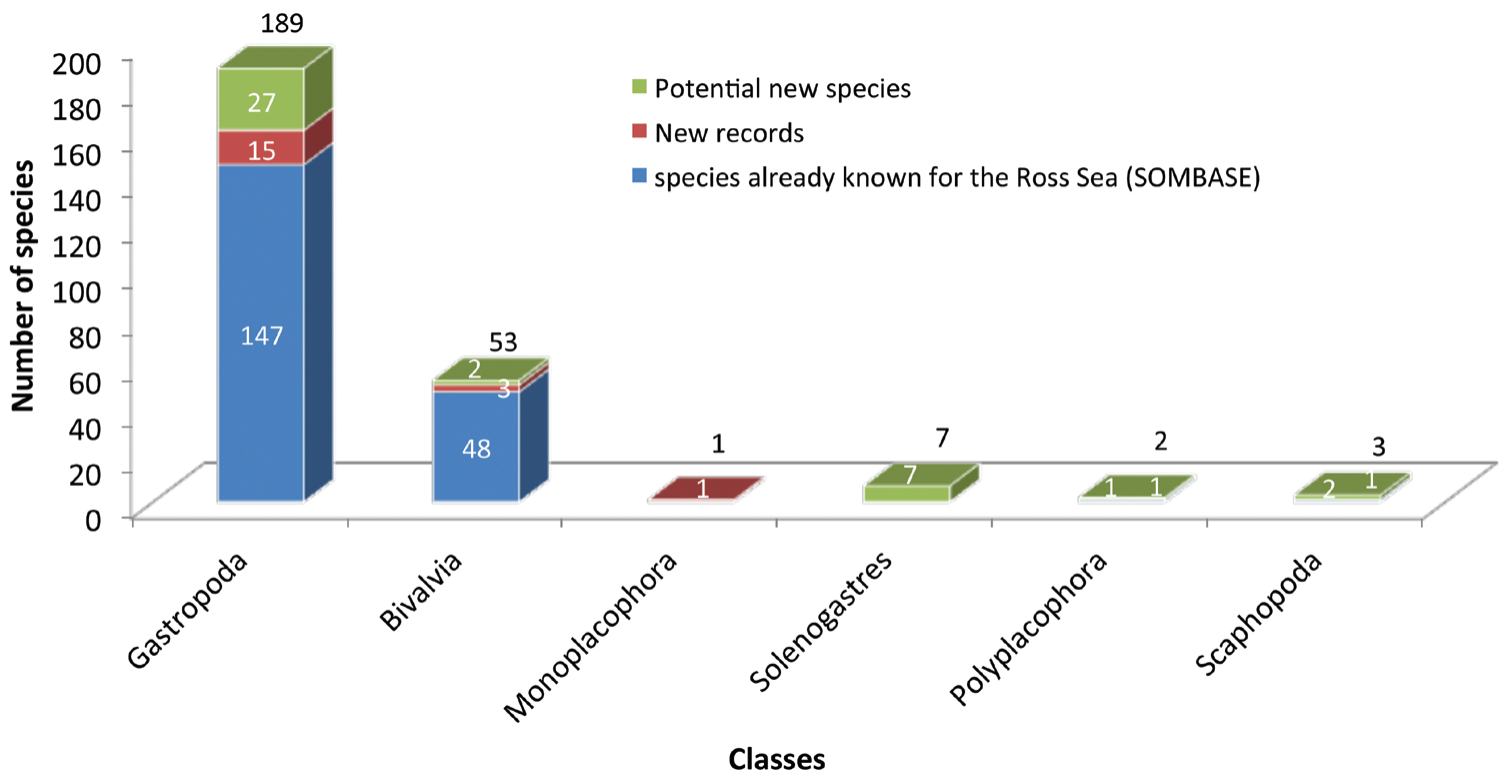
Number of new species records compared to the available baseline (SOMBASE, www.antarctica.ac.uk/sombase). In light blue: species already known for the Ross Sea; in red: new record of know species never found before in the Ross Sea; in green: species classified at the generic level only a potentially representing new species for the area.

**General taxonomic coverage description:** The present dataset focuses on the Kingdom Animalia, Phylum Mollusca and includes six molluscs classes: Gastropoda, Bivalvia, Monoplacophora, Solenogastres, Polyplacophora and Scaphopoda. In total 8,359 specimens have been collected belonging to 161 species and corresponding to 505 species distributional records. Of these, in order of abundance, 5,965 specimens were Gastropoda (accounting for 113 species), 1,323 were Bivalvia (accounting for 36 species), 949 were Aplacophora (accounting for 7 species), 74 specimens were Scaphopoda (3 species), 38 were Monoplacophora (1 species) and, finally, 10 specimens were Polyplacophora (1 species). This data set represent the first large-scale survey of benthic micromolluscs for the area and provides important information about the distribution of several species which have been seldom or never recorded before in the Ross Sea. A detailed analysis of the distribution of mollusc species sampled by the Rauschert dredge as well as the illustration of all new records for the Ross Sea is in Ghiglione et al. (submitted). The number of newly reported species for the Ross Sea is compared with the available base line, i.e. the SOMBASE records, in Figure 3.

The dataset includes respectively for each class:

**Kingdom:** Animalia

**Phylum:** Mollusca

**Class**: Solenogastres

**Family:** Neomeniidae

**Species:** Solenogastres sp. 1, Solenogastres sp. 2, Solenogastres sp. 3, Solenogastres sp. 4, Solenogastres sp. 5, Solenogastres sp. 6, *Neomenia* sp. 1

**Kingdom:** Animalia

**Phylum:** Mollusca

**Class:** Polyplacophora

**Family:** Callochitonidae

**Genus:** *Callochiton*

**Species:** *Callochiton* sp. 1

**Kingdom:** Animalia

**Phylum:** Mollusca

**Class:** Monoplacophora

**Family:** Micropilinidae

**Genus:** *Micropilina*

**Species:** *Micropilina arntzi* Warén & Hain, 1992

**Kingdom:** Animalia

**Phylum:** Mollusca

**Class:** Gastropoda

**Family:** Acteonidae, Cancellaridae, Anatomidae, Margaritidae, Doridae, Eulimidae, Mangeliidae, Seguenzioidea, Calliotropidae, Capulidae, Newtoniellidae, Buccinidae, Skeneidae, Cylichnidae, Diaphanidae, Dotidae, Eatoniellidae, Zerotulidae, Lepetidae, Naticidae, Cerithiopsidae, Collonidae, Skeneidae, Mangellidae, Margaritidae, Orbitestellidae, Triviidae, Omalogyridae, Volutomitridae, Buccinidae, Philinidae, Raphitomidae, Rissoidae, Akiodorididae, Cingulopsidae, Pyramidellidae, Tjaernoeiidae, Muricidae, Mathildidae, Borsoniidae

**Genus:** *Acteon*, *Admete*, *Aegires*, *Anatoma*, *Antimargarita*, *Antistreptus*, *Austrodoris*, *Bathycrinicola*, *Belalora*, *Brookula*, *Calliotropis*, *Capulus*, *Cerithiella*, *Chlanidota*, *Cirsonella*, *Cylichna*, *Diaphana*, *Doto*, *Eatoniella*, *Eumetula*, *Frovina*, *Fusceulima*, *Haliella*, *Hemiaclis*, *Iothia*, *Kerguelenatica*, *Krachia*, *Leptocollonia*, *Liotella*, *Lissotesta*, *Lorabela*, *Margarites*, *Melanella*, *Microdiscula*, *Newnesia*, *Nothoadmete*, *Notoficula*, *Omalogyra*, *Paradmete*, *Pareuthria*, *Philine*, *Pleurotomella*, *Powellisetia*, *Probuccinum*, *Prodoridunculus*, *Prosipho*, *Sinuber*, *Skenella*, *Streptocionella*, *Thjaernoeia*, *Toledonia*, *Torellia*, *Trilirata*, *Trophon*, *Turritellopsis*, *Typhlodaphne*

**Species:** *Acteon antarcticus* Thiele, 1912, *Admete haini* Numanami, 1996, *Aegires albus* Thiele, 1912, *Anatoma euglypta* (Pelseneer, 1903), *Antimargarita dulcis* (E. A. Smith, 1907), *Doris kerguelensis* (Bergh, 1884), *Bathycrinicola tumidula* (Thiele, 1912), *Oneopota striatula* (Thiele, 1912), *Bertellidae* sp.1, *Bertellidae* sp.2, *Brookula pfefferi* Powell, 1951, *Brookula* cf. *argentina* Zelaya, Absalao & Pimienta, 2006, *Brookula strebeli* A.W.B. Powell, 1951, *Calliotropis antarctica* Dell, 1990, *Cancellaridae* sp.1, *Cancellaridae* sp.2, *Capulus subcompressus* Pelseneer, 1903, *Cerithiella seymouriana* (Strebel, 1908), *Chlanidota signeyana* A.W.B. Powell, 1951, *Cirsonella extrema* Thiele, 1912, *Cylichna gelida* (E. A. Smith, 1907), *Diaphana paessleri* (Strebel, 1905), *Doto antarctica* Eliot, 1907, *Doto* sp., *Eatoniella* aff. *cana* Ponder, 1983, *Eatoniella* cf. *demissa* (E. A. Smith, 1915), *Eatoniella kerguelensis* (E. A. Smith, 1875), *Eulimidae* sp.1, *Eulimidae* sp.2, *Eulimidae* sp.3, *Eulimidae* sp.4, *Eulimidae* sp.5, *Eumetula dilecta* (Thiele, 1912), *Eumetula* cf. *dilecta* (Thiele, 1912), *Eumetula strebeli* (Thiele, 1912), *Frovina* sp.1, *Frovina* sp.2, *Fusceulima* sp.1, *Fusceulima* sp.2, *Gastropoda* sp.1, *Haliella* sp.1, *Hemiaclis incolorata* (Thiele, 1912), *Hemiaclis* sp.1, *Hemiaclis* sp.2, *Iothia emarginuloides* (Philippi, 1868), *Kerguelenatica delicatula* (E. A. Smith, 1902), *Krachia antartica* (E. A. Smith, 1907), *Leptocollonia innocens* (Thiele, 1912), *Liotella* sp.1, *Lissotesta macknighti* (Dell, 1990), *Lissotesta mammillata* (Thiele, 1912), *Lissotesta minutissima* (E. A. Smith, 1907), *Lissotesta notalis* (Strebel, 1908), *Lissotesta similis* (Thiele, 1912), *Lissotesta* sp.1, *Lissotesta strebeli* (Thiele, 1912), *Lissotesta unifilosa* (Thiele, 1912), *Lorabela davisi* (Hedley, 1916), *Margarites crebrilirulata* (E. A. Smith, 1907), *Margarites refulgens* (E. A. Smith, 1907), *Marseniopsis* sp., *Melanella antarctica* (Strebel, 1908), *Melanella convexa* (E. A. Smith, 1907), *Microdiscula vanhoeffeni* Thiele, 1912, *Naticidae* sp.1, *Newnesia antartica* E. A. Smith, 1902, *Nothoadmete* cf. *delicatula* (E. A. Smith, 1907), *Notoficula bouveti* (Thiele, 1912), *Omalogyra burdwoodiana* Strebel, 1908, *Omalogyra* sp.1, *Onoba egorovae* Numanami, 1996, *Onoba gelida* (E. A. Smith, 1907), *Onoba kergueleni* (E. A. Smith, 1875), *Onoba paucilirata* (Melvill & Standen, 1912), *Onoba* sp.1, *Onoba subantarctica wilkesiana* (Hedley, 1916), *Onoba turqueti* (Lamy, 1905), *Paradmete fragillima* (Watson, 1882), *Pareuthria plicatula* Thiele, 1912, *Philine alata* Thiele, 1912, *Pleurotomella deliciosa* Thiele, 1912, *Powellisetia deserta* (E. A. Smith, 1907), *Probuccinum tenerum* (E. A. Smith, 1907), *Prodoridunculus gaussianus* Thiele, 1912, *Prosipho nodosus* Thiele, 1912, *Antistreptus contrarius* (Thiele, 1912), *Prosipho glacialis* Thiele, 1912,, *Prosipho mundus* E. A. Smith, 1915, *Sinuber microstriatum* Dell, 1990, *Skenella paludinoides* (E. A. Smith, 1902), *Streptocionella pluralis* Dell, 1990, *Tjaernoeia michaeli* Engl, 2002, *Toledonia* cf. *perplexa* Dall, 1902, *Toledonia elata* Thiele, 1912, *Toledonia globosa* Hedley, 1916, *Toledonia limnaeaeformis* (E. A. Smith, 1879), *Toledonia major* (Hedley, 1911), *Toledonia palmeri* Dell, 1990, *Toledonia punctata* Thiele, 1912, *Toledonia* sp.1, *Toledonia* sp.2, *Toledonia* sp.3, *Toledonia striata* Thiele, 1912, *Torellia antarctica* (Thiele, 1912), *Torellia exilis* (Powell, 1958), *Trilirata macmurdensis* (Hedley, 1911), *Trilirata sexcarinata* Warén & Hain, 1996, *Trophon coulmanensis* E. A. Smith, 1907, *Trophon minutus* Melvill & Standen, 1907, *Turritellopsis latior* Thiele, 1912, *Typhlodaphne innocentia* Dell, 1990, *Typhlodaphne* sp.1

**Kingdom:** Animalia

**Phylum:** Mollusca

**Class:** Bivalvia

**Family:** Philobryidae, Astartidae, Cuspidariidae, Cyamiidae, Carditidae, Cyclochlamydidae, Propeamussiidae, Mytilidae, Kelliidae, Limidae, Limopsidae, Philibryidae, Lyonsiidae, Montacutidae, Poromyidae, Nuculanidae, Siliculidae, Cuspidariidae, Thraciidae, Thyasiridae, Galeommatoidea, Yoldiidae

**Genus:** *Adacnarca*, *Astarte*, *Cuspidaria*, *Cyamiomactra*, *Cyclocardia*, *Cyclochlamys*, *Cyclopecten*, *Dacrydium*, *Kellia*, *Limatula*, *Limopsis*, *Lissarca*, *Lyonsia*, *Montacuta*, *Mysella*, *Parathyasira*, *Philobrya*, *Poromya*, *Propeleda*, *Pseudokellya*, *Silicula*, *Subcuspidaria*, *Thracia*, *Waldo*, *Yoldiella*

**Species:** *Adacnarca nitens* Pelseneer, 1903, *Astarte longirostris* d’Orbigny, 1842, *Cuspidaria tenella* E. A. Smith, 1907, *Cuspidaria kerguelensis* (E. A. Smith, 1885), *Cyamiomactra laminifera* (Lamy, 1906), *Cyamiomactra robusta* Nicol, 1964, *Cyclocardia astartoides* (Martens, 1878), *Cyclochlamys gaussiana* (Thiele, 1912), *Cyclochlamys pteriola* (Melvill & Standen, 1907), *Dacrydium albidum* Pelseneer, 1903, *Kellia simulans* E. A. Smith, 1907, *Limatula hodgsoni* (E. A. Smith, 1907), *Limatula ovalis* (Thiele, 1912), *Limatula simillima* (Thiele, 1912), *Limopsis lilliei* E. A. Smith, 1915, *Limopsis marionensis* E. A. Smith, 1885, *Lissarca notorcadensis* Melvill & Standen, 1907, *Lyonsia arcaeformis* Martens, 1885, *Montacuta nimrodiana* Hedley, 1911, *Mysella* cf. *antarctica* (E. A. Smith, 1907), *Mysella charcoti* Lamy, 1906, *Mysella gibbosa* (Thiele, 1912), *Mysella* sp.1, *Philobrya sublaevis* Pelseneer, 1903, *Philobrya wandelensis* Lamy, 1906, *Philobrydae* sp.1, *Poromya spinosula* Thiele, 1912, *Propeleda longicaudata* (Thiele, 1912), *Pseudokellya gradate* Thiele, 1912, *Pseudokellya* sp. juv., *Silicula rouchi* Lamy, 1911, *Thracia meridionalis* E. A. Smith, 1885, *Parathyasira dearborni* (Nicol, 1965), *Thyasira debilis* (Thiele, 1912), *Waldo parasiticus* (Dall, 1876), *Yoldiella antarctica* (Thiele, 1912)

**Kingdom:** Animalia

**Phylum:** Mollusca

**Class:** Scaphopoda

**Family:** Pulsellidae, Gadilidae

**Genus:** *Pulsellum*, *Siphonodentalium*

**Species:** *Pulsellum* sp. 1, *Siphonodentalium dalli* (Pilsbry & Sharp, 1898), *Siphonodentalium* sp. 1

## Spatial coverage

### General spatial coverage

Ross Sea, Antarctica (Figure 2).

### Coordinates

71°15'5"S and 74°49'3"S Latitude; 164°11'5"E and 170°41'9"E Longitude.

### Temporal coverage

February 9, 2004–February 21, 2004.

## Natural collections description

**Parent collection identifier:** Italian Antarctic National Museum (Section of Genoa, Italy)

**Collection name:** Italica 2004 Rauschert Molluscs

**Collection identifier:** http://www.mna.it

**Specimen preservation method:** Specimens were fixed in pre-cooled Ethanol immediately after the extraction from the dredge net. In this way any thermal shock which could potentially alter the integrity of DNA was avoided. After fixation, specimens were sorted under a stereomicroscope, divided into morphospecies and stored in „Screw Thread Vials“ (National Scientific, USA). For study, some specimens per species have been dissected under the stereomicroscope and soft parts used for DNA extractions. Shells corresponding to these specimens have been dried in increasing ethanol concentrations, mounted on stubs and gold sputtered for scanning electron microscope observation. These specimens are maintained in a laboratory kiln with silica gel to prevent deterioration. All the other specimens are kept in ethanol in the collections of the Italian National Antarctic Museum.

## Datasets

### Dataset description

This dataset contains data about the Phylum Mollusca in the Ross Sea. In particular, it includes 161 species for a total of 8,359 specimens. By cconsidering this dataset in terms of incidence, it encompasses 505 discrete distributional records.

The Darwin Core elements included in the dataset are: scientific name, collection code (i.e. MNA acronym), catalogue number (i.e. MNA catalogue number), year of collection, date of collection, latitude and longitude (in decimal degrees), individual counts, and basis of records (type of preservation).

**Object name:** Italica 2004_Rauschert dredge_Ross_sea_Mollusca_lgp

**Character encoding:** UTF-8

**Format name:** Darwin Core Archive format

**Format version:** 1.0

**Distribution:** http://ipt.biodiversity.aq/resource.do?r=ross_sea_mollusca_lgp

**Language:** English

**Metadata language:** English

**License of use**: This dataset [Italica 2004_Rauschert dredge_Ross_sea_Mollusca_lgp] is made available under the Open Data Commons Attribution License: http://www.opendatacommons.org/licenses/by/1.0/

**Date of metadata creation:** 2013-01-08

**Hierarchy level:** Dataset
